# High fidelity epigenetic inheritance: Information theoretic model predicts threshold filling of histone modifications post replication

**DOI:** 10.1371/journal.pcbi.1009861

**Published:** 2022-02-17

**Authors:** Nithya Ramakrishnan, Sibi Raj B. Pillai, Ranjith Padinhateeri

**Affiliations:** 1 Department of Biosciences and Bioengineering, Indian Institute of Technology Bombay, Mumbai, India; 2 Department of Electrical Engineering, Indian Institute of Technology Bombay, Mumbai, India; University of Essex, UNITED KINGDOM

## Abstract

During cell devision, maintaining the epigenetic information encoded in histone modification patterns is crucial for survival and identity of cells. The faithful inheritance of the histone marks from the parental to the daughter strands is a puzzle, given that each strand gets only half of the parental nucleosomes. Mapping DNA replication and reconstruction of modifications to equivalent problems in communication of information, we ask how well enzymes can recover the parental modifications, if they were ideal computing machines. Studying a parameter regime where realistic enzymes can function, our analysis predicts that enzymes may implement a critical threshold filling algorithm which fills unmodified regions of length at most *k*. This algorithm, motivated from communication theory, is derived from the maximum à posteriori probability (MAP) decoding which identifies the most probable modification sequence based on available observations. Simulations using our method produce modification patterns similar to what has been observed in recent experiments. We also show that our results can be naturally extended to explain inheritance of spatially distinct antagonistic modifications.

## Author summary

Chromatin is essentially the DNA that is folded and packaged with the help of proteins. While the nucleotide sequence in the DNA codes genetic information, the packaging of the DNA into chromatin encodes extra layer of information. This epigenetic code regulates reading of the genetic code and provides identity to cells—whether the cell is a skin cell or a brain cell, for example. In this work we examine a long standing puzzle, that is, how the epigenetic code in the form of histone modification patterns may get inherited, when a cell divides. Using theoretical arguments, we present an algorithm that enzymes could be executing so that the epigenetic code can be inherited with minimal error, soon after DNA replication.

## Introduction

**Fig 1:**
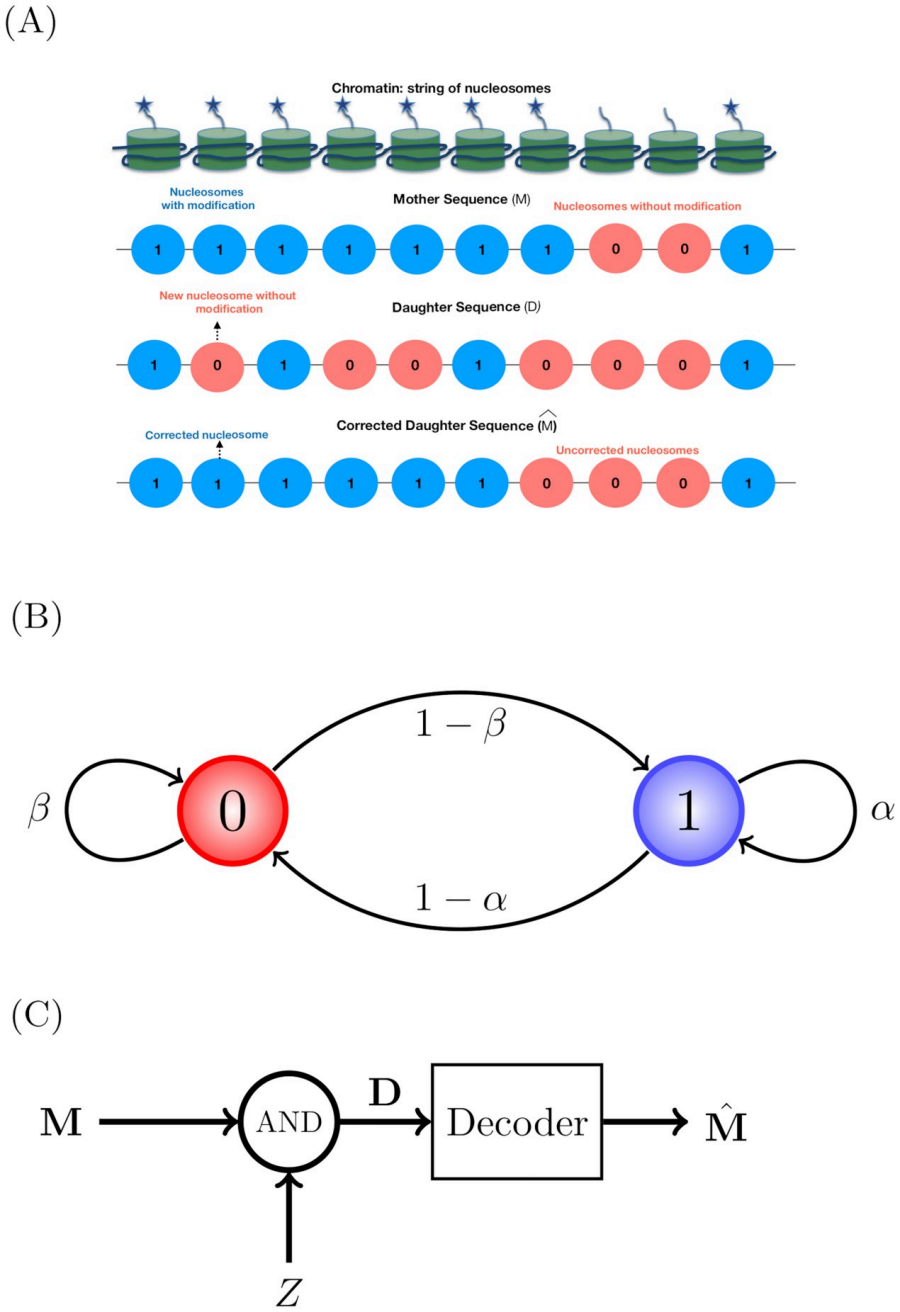
Schematic description of the problem (A) Row 1 from top: chromatin as a string of nucleosomes with and without the histone modification (star) of our interest. This can be mapped to a string of binary numbers indicating the presence (1) or absence (0) of the modification (row 2), giving us **M**. Row 3: one typical realization of a daughter chromatin **D** produced from **M** above, via a process mimicking DNA replication, where only a fraction of the modifications (1s) will end up in the daughter chromatin, stochastically; the rest do not have the modification of interest (0) [25]. Post replication, certain enzymes will insert modifications correcting **D** to a mother-like sequence $\hat{\text{M}}$ (row 4). Since these are stochastic processes, we expect some errors. (B) The mother sequence (**M**) is modeled as a first order Markov chain having sequence of 0s and 1s. *α* and *β* are probabilities of finding a 1 followed by a 1, and a 0 followed by a 0, respectively. 1 − *α* and 1 − *β* are probabilities of finding a 1 followed by a 0 (note arrowheads), and a 0 followed by a 1, respectively. (C) The daughter sequence **D** is obtained by a mother sequence **M** getting logically ANDed with an independent and identically distributed (IID) binary sequence **Z** (noise). A mother-like sequence $\hat{M}$ is reconstructed by passing **D** through a decoder. The plausible ways by which enzymes could act as decoders is the subject of this study.

Why do our bone cells behave very differently from our muscle cells or cells of other types even though they all have the same genetic code? To explain the emergence of such diverse cell types, one would need to note that, beyond the genetic code, there are multiple layers of information encoded by the wrapping and folding of the DNA into chromatin with the help of many proteins [1, 2]. Most of the DNA is wrapped around octamers of histone proteins, making the chromatin, essentially, like a string of beads made of nucleosomes (DNA+histones) [3–6]. Each nucleosome carries chemical modifications, like acetylations and methylations [7], forming a pattern of histone marks along the chromatin polymer contour [2, 8–11] (see Fig 1A top panel). This pattern encodes a crucial layer of information regulating accessibility, transcription, replication and other cellular processes [11]. Even though the entire histone modification code is not deciphered yet, we understand it in parts. For example, H3K27me3/H3K9me3 represses reading of the local DNA region where the modification is present, H3K9ac/H3K4me3 encodes for local gene activation and so on [7, 12, 13]. The activation and repression of genes collectively decide the gene expression pattern and hence determine the function and fate of a cell [10, 14–16].

While preparing to divide, cells copy their genetic code via the DNA replication process. For DNA to be copied, the chromatin has to be unfolded and histone proteins need to be disassembled [17, 18]. This would disrupt the pattern of histone modifications. Recent studies have shown that the (H3 − H4)_2_ tetramer from the parent remains intact [19] and randomly gets deposited onto either of the newly synthesized DNA strands [20–22]. That is, daughter strands will have only some (≈ 50%) of its nucleosomes from the parent; the rest need to be assembled from the pool of new histone proteins made afresh [23, 24]. Since the new nucleosomes will not carry the histone marks present on the parental chromatin, half the parental marks are missing in each daughter chromatin [25]. Since the histone modification patterns can decide the state (repressed/active) of all genes and the identity of the cell itself, it is crucial that the newly made chromatin re-establishes the pattern immediately after replication [26]. Recent experiments show that many of the histone modification patterns—patterns in the repressed gene regions, in particular—are “inherited” from the parental chromatin [27–31]. We know from literature that the heterochromatin region is inherited faithfully during both mitosis and meiosis [27, 32, 33]. It is known that there are specific enzymes to read and write histone marks [31, 33–35]. While molecular details of some of these enzymes are known [36–38], precisely what strategies they use to re-establish the histone modification pattern after replication are not fully understood.

Previous theoretical studies have investigated average properties of modifications and whether long-range interactions are necessary or short-range interactions would suffice [39, 40]. Dodd et al [40] present a stochastic model and investigate the bistability similar to what is observed in silent mating-type region in *S. pombe*. This model has been further extended to discuss the likelihood of bivalency and bistability in poised chromatin [41]. Additionally, there have also been various models exploring the maintenance of chromatin state in specific types of cells [42–44]. Another set of models investigated establishment of stable modification patterns near a nucleation site [45, 46]. The antagonism between certain modification pairs, leading to domains in the chromatin, have also been quantitatively explored [47]. Several other studies have discussed the possible nature of the spread of the histone modifications and maintenance of the chromatin states [48–52]. However, all aspects of the experimentally observed [28] high-fidelity re-establishment of the modification patterns post replication along the genome (spatial pattern) is not explained by the existing models.

In the past, information theory has been applied to biological systems to study several aspects of gene regulation and signalling [53–55]. The problem of loss of information in the modification pattern during replication and its retrieval within the daughter chromatin is very similar to data loss and error correction in telecommunication. In such systems, a transmitted signal gets exposed to noise and consequently becomes, error-prone at the receiving end. The decoder at the receiver detects and corrects these errors using techniques from information and communication theory [56, 57]. This viewpoint immediately poses the following questions: Can we use known decoding algorithms from communication theory to analyze chromatin modification loss and retrieval? How well can the best known algorithms correct the missing modifications and re-establish the modification patterns? What is the best possible correction strategy enzymes could use if they were ideal computing machines? Are these algorithms compatible with the biological processes that realistic cellular enzymes can conceivably do? In this paper, we address these questions using ideas from information theory. We consider one of the daughter chromatins to be a noise-corrupted signal created at the replication fork, while the enzymes and other molecular agents help to correct this error using mathematical techniques. In this model, the inheritance of the mother’s pattern is approached using Bayesian decoding techniques. We show that our model can reconstruct histone modification patterns present in publicly available experimental data [28], suggesting that the model is relevant.

## Model and methods

Consider a region on a mother chromatin having *N* nucleosomes. We are interested in studying the inheritance of one histone modification at a time. Since many of the repressive marks are known to be inherited accurately [28] after replication, we will consider one such repressive mark (e.g., H3K27me3) and its pattern along a chromatin. This pattern can be represented by a vector **M** = (*m*_1_, *m*_2_, … *m*_*N*_), where *m*_*i*_ can take values 1 or 0 indicating the presence or absence of the modification on the *i*^*th*^ nucleosome (see Fig 1A).

For brevity, we use the following notations:

-
$m_{i}^{j}$ represents the sequence of modification values (*m*_*i*_, *m*_*i*+1_, …, *m*_*j*_) between location *i* and *j* with *j* > *i*; Thus, $M=m_{1}^{N}$ represents the entire mother chromatin modification sequence.
- a region with *k* consecutive modifications present (ones) or absent (zeros) will be denoted as 1_*k*_ or (0_*k*_), respectively. Extending this, the sequence (1, 0, …, 0, 1) representing an island of *k* consecutive zeros between two ones will be denoted as (1, 0_*k*_, 1).

Models with nearest neighbor interactions (Markov model of order 1) are commonly used to represent the genetic/epigenetic sequences and other biophysical phenomena like protein-binding [58]. For instance, Zhang et al [39] have used a nearest neighbor interaction model to study epigenetic memory. The modeling of experimental data by Hodges and Crabtree [45] also assumes that each nucleosome interacts with only its nearest neighbour and that the modification is passed on to the nearest neighbour with a certain probability. Poepsel et al [59] show that histone methyl transferase protein PRC2 simultaneously engages with two nucleosomes—one unmodified nucleosome and one H3K27me3-containing nucleosome—suggesting a modification spreading mechanism among neighbouring nucleosomes. Many of the existing models have the underlying assumption of nearest neighbor interactions (Markov model of order 1). In our work, we make a similar assumption: Since modification on a nucleosome is very likely related to its immediate neighbors, we model the pattern **M** along the mother chromatin as a binary-valued random walk, having neighbourhood interactions corresponding to a first order homogeneous Markov chain. More specifically, given the modifications *m*_*i*−1_ and *m*_*i*+1_, the modification *m*_*i*_ is assumed to be independent of all other modification values. Equivalently, the conditional probability law is

$$\begin{array}{c} P\left(m_{i}|m_{1},\ldots ,m_{i-1}\right)=P\left(m_{i}|m_{i-1}\right)\;,\;\;i\geq 2, \end{array}$$ (1)

where *m*_1_ is the modification on the first nucleosome of the region of our interest. This is also similar to the 1-dimensional Ising model where the interaction energy of the *i*^th^ site is coupled only to the immediate neighboring sites [60, 61]. The state-space evolution of the Markov chain **M** is as follows: given *m*_*i*_ = 1, let *α* and 1 − *α* be the probabilities for obtaining *m*_*i*+1_ = 1 and *m*_*i*+1_ = 0 respectively. Similarly, if *m*_*i*_ = 0, let *β* and 1 − *β* be the probabilities for having *m*_*i*+1_ = 0 and *m*_*i*+1_ = 1 respectively. The sequence **M** can be seen as a random walk on the state space shown in Fig 1B. The parameters *α* and *β* are functions of the mean contiguous length of the modified and unmodified regions respectively and can be computed from the experimental data. For example, when *α* and *β* values are close to 1, the pattern would often contain long runs of either 1s (presence of modification) or 0s (absence of modification). See S1 and S2 Text.

From the mother chromatin **M**, the generation of a daughter chromatin having histone modification sequence $D=d_{1}^{N}$ is modeled as follows. During replication, with probability $\frac{1}{2}$, each nucleosome on a daughter chromatin is either directly inherited from its parental counterpart (i.e. *d*_*i*_ = *m*_*i*_) or newly deposited (i.e. *d*_*i*_ = 0) from a pool of fresh histones assembled de novo [25]. We consider both the histones in the (*H*3 − *H*4)_2_ tetramer to be symmetrically modified in our model based, on recent evidence that a large proportion of the histones are symmetrically modified [19, 62]. This process is equivalent to doing a logical AND operation of the mother sequence **M** with an independent binary vector **Z** (noise), which is generated by independent tosses of a fair coin. Thus, *d*_*i*_ = *m*_*i*_ ⋅ *z*_*i*_, 1 ≤ *i* ≤ *N* (see Fig 1C), where **Z** = (*z*_1_, *z*_2_, *z*_3_, …, *z*_*N*_) has Independent and Identically Distributed (IID) entries. This biological process leads to a memoryless model with the conditional probability

$$\begin{array}{c} P\left(d_{1}^{N}|m_{1}^{N}\right)=\prod_{i=1}^{N}P\left(d_{i}|m_{i}\right). \end{array}$$ (2)

Owing to the independent and random nature of the placement of the parental nucleosomes along the newly formed daughter chromatin, the above relation is justified. In biology, the question is, given a daughter sequence $d_{1}^{N}$ post replication, how can a cell reconstruct a mother-like sequence $\hat{M}={\hat{m}}_{1}^{N}$? In other words, is it possible to build a decoder that would reconstruct a $\hat{M}$ from **D**, as depicted in Fig 1C? Ideally, a cell would want to choose a binary sequence $\hat{M}$ having the minimum deviation from **M**. A simple way to quantify this deviation is to compute the fraction of errors in the reconstructed sequence as:

$$\begin{array}{c} \Delta \left(M,\hat{M}\right)=\frac{1}{N}\sum_{i=1}^{N}{\left(m_{i}-{\hat{m}}_{i}\right)}^{2}. \end{array}$$ (3)

Since we are comparing bitwise, this deviation metric is effectively the bit error rate (BER) when *N* becomes large [63]. In communication, BER is the proportion of bits that are in error among the total transmitted/received bits. In the present context, BER is essentially the fraction of nucleosomes in a daughter sequence that differ in their histone mark from the parental sequence. Thus the chosen $\hat{M}$ should minimize the BER with respect to the actual sequence **M**, while obeying the transition law in Eq (1). This is similar to data communication through an erroneous channel. It is well known that Bayesian estimation schemes minimize the average detection error probability at the receiver. In particular, a decoder choosing the input sequence having the Maximum À posteriori Probability (MAP) is optimal in minimizing the message error probability in communication [57, 63] (see S1 Text). We call this the Sequence MAP (SMAP) decoder, which identifies the most probable sequence $\hat{M}=\left({\hat{m}}_{1},\ldots ,{\hat{m}}_{N}\right)$ based on the observations $d_{1}^{N}$ as

$$\begin{array}{c} \left({\hat{m}}_{1},\ldots ,{\hat{m}}_{N}\right)=\underset{m_{1},\ldots ,m_{N}}{\text{argmax}}P\left(m_{1}^{N}|d_{1}^{N}\right). \end{array}$$ (4)

The above equation essentially says that, given a daughter sequence, the sequence that maximizes the conditional probability should be declared as the reconstructed mother-like sequence. SMAP decoding is known to have very good BER performance and good analytical tractability in many contexts [63]. Though SMAP decoding is not directly minimizing BER, it nevertheless is known to have good BER performance as well.

**Implementation of SMAP algorithm**: To implement the SMAP algorithm, one has to maximize the conditional probability P(M|D) over possible sequences of **M**, based on the given daughter sequence observations $D=d_{1}^{n}$. Applying Bayes’ rule [64], along with Eqs (1) and (2) (see S1 Text) one gets

$$\begin{array}{c} P\left(M|D\right)=\frac{1}{P(D)}\prod_{i=1}^{N}P\left(m_{i}|m_{i-1}\right)P\left(d_{i}|m_{i}\right), \end{array}$$ (5)

where we took *m*_0_ = ∅ (empty set) for notational convenience. Since P(D) does not play a role in the maximization over **M**, it can be ignored for our purposes. The equation says that the reconstruction process of the mother-like sequence needs to account for two facts: the modification status of the immediate neighbours ($P(m_{i}|m_{i-1})$) and the information embedded in the replication process ($P(d_{i}|m_{i})$). Knowing Eq (5), ideal computing machines can now implement the SMAP algorithm using the idea of trellis decoding [56], which is closely related to the well known Viterbi Algorithm in coding theory [63]. While the memory and computational power requirement for trellis decoding is high in general, we find that decoding procedure for our model can be broken down to smaller sub-sequences, each corresponding to a different run of zeros in **D**. In particular, SMAP decoding can be applied separately on sub-sequences of the form (1, 0_*k*_, 1), which has *k* consecutive zeros in between two ones. Mathematically, we can show that SMAP decoding will choose a sequence having ${\hat{m}}_{i}^{j}$ according to ${\hat{m}}_{i},\ldots ,{\hat{m}}_{j}=\text{argmax}\;P\left(m_{i},\ldots ,m_{j}|d_{i}=1,d_{i+1}^{j-1},d_{j}=1\right)$; here *i* and *j* be two positions (with *j* > *i*) where the daughter sequence has ones (see S1 and S2 Text. Our analysis suggests that to decide on a bit at position *l* where the daughter has inherited a zero, we need to only consider the smallest daughter segment containing the position *l*, and flanked by ones at both ends. Only daughter segments with at least one intermediate zero are to be considered; otherwise there is nothing to decode. Without loss of generality, we will take **D** = (1, 0_*k*_, 1) for the rest of the exposition, corresponding to a run of *k* zeros, and perform trellis decoding on this sequence.

**Fig 2:**
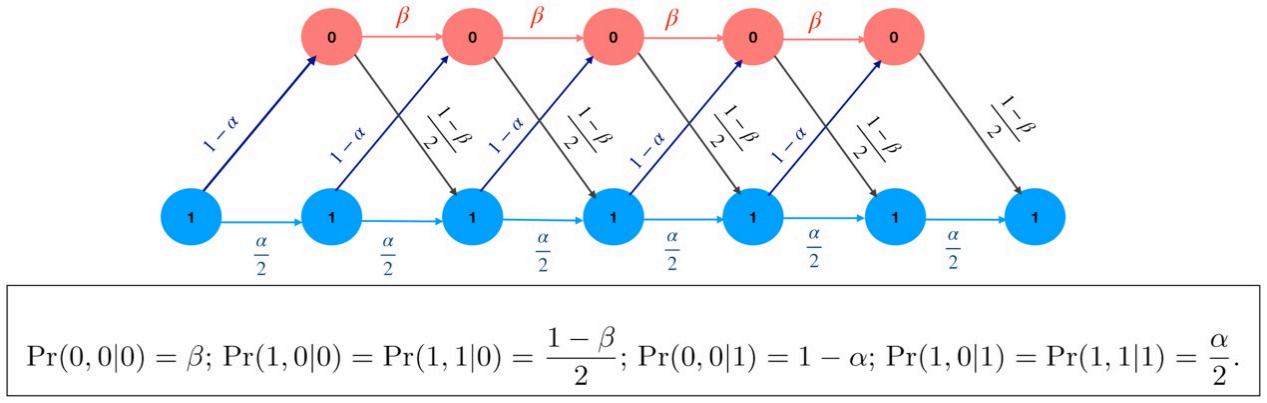
The trellis diagram used for illustrating how the MAP algorithm chooses the path of maximum a posteriori probability given a daughter sequence (1, 0_5_, 1). From each Markov state (0 or 1), there are two possible arrows to transition to the next state. The values $P(m_{i},d_{i}|m_{i-1})$ given in the box represent probabilities associated with each arrow. Starting from state 1 at the left bottom, for each possible path moving along the arrows, one can compute the path probabilities (path metrics) using Eq (5). We finally choose the path with the largest path metric. This is based on the Viterbi algorithm in communication theory.

**Trellis Decoding**: Trellis decoding [56] is a technique that can identify the most probable mother-like histone modification sequence, according to the theory described above. In this study, a sequence of states of a Markov chain (here, histone modification sequence) is called a path or a trajectory. Given the states at the start and end of a possible path, a trellis diagram [56] can be used to find the joint probability of each path with the given observation **D**. Since **D** has the form (1, 0_*k*_, 1), the start and end states of the trellis are ones. Fig 2 demonstrates the trellis diagram for a run of 5 zeros (*k* = 5). Starting from the initial state 1 (left bottom in Fig 2), the trellis diagram assigns a conditional probability (branch metric) to each subsequent transition (arrow), based on the transition probability law and observed daughter state. For transitions from state at *i* − 1 to *i*, the branch metric is $P(m_{i},d_{i}|m_{i-1})$, which can be evaluated as $P\left(m_{i}|m_{i-1}\right)P\left(d_{i}|m_{i}\right)$, where *d*_*i*_ = 0 for 2 ≤ *i* ≤ *N* − 1. While the sequence **D** is easily seen to be generated by a hidden Markov Model (HMM), the branch probability metrics are explicitly given inside the box of Fig 2 (also see S1 Text). Notice that we have to find $P(m_{i},d_{i}=0|m_{i-1})$ for (*m*_*i*−1_, *m*_*i*_) ∈ {(0, 0), (0, 1), (1, 0), (1, 1)}, and $P(m_{i}=1,d_{i}=1|m_{i-1})$ for *m*_*i*−1_ ∈ {0, 1} for the last stage (see Fig 2). The computation of the branch and path metrics and the choice of the path with the largest path metric is performed using the Viterbi Algorithm in communication theory [56].

Notice that each possible mother sequence can be identified as a path in the trellis, with the labels identifying the branch metrics. Using Eq (5), the product of corresponding branch metrics will yield the path metric of each possible sequence, and then the path maximizing the SMAP metric can be chosen.

## Results

In this section, we will be using the ideas developed in the Model and Methods to answer how one can reconstruct a mother-like modification sequence $\hat{M}$, given the daughter chromatin sequence **D**. We will discuss how well algorithms like the Sequence MAP (SMAP) decoding will compute $\hat{M}$, and whether realistic enzymes can implement this in practice.

### Ideal enzymes implementing SMAP decoding

From recent experiments, it has been established that a plethora of enzymes and histone chaperones (including FACT, MCM2) are involved in the inheritance of the histone modifications post replication [20, 21, 31]. Yet, how the enzyme machinery uses the parental information and tries to reproduce the exact pattern of the histone modification, post-replication, is not clear. In this work, we discuss how well can the communication theory-inspired algorithms reconstruct a mother-like sequence. To test this, one can imagine some *ideal enzymes* —computing machines— constructed to implement the SMAP algorithm in Eq (4); they will achieve this by maximizing the conditional probability P(M|D) as mentioned above.

**Fig 3:**
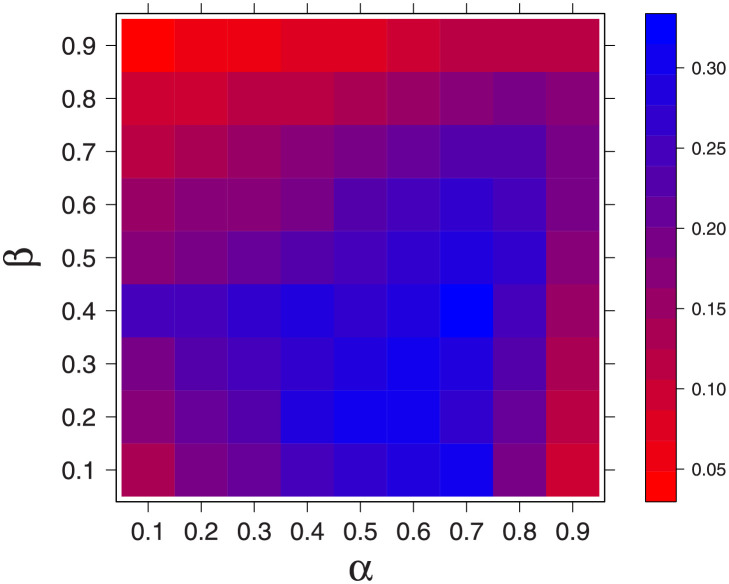
The average deviation between the original mother and the mother-like corrected daughter sequences ($\bar{\Delta }=$ ensemble averaged $\Delta (M,\hat{M})$) is plotted for different *α* and *β* values as a heatmap (see color bar on the side). The error is averaged over the error of 300 mother sequences and 200 daughter sequences corresponding to each mother sequence—that is, 60000 $\Delta (M,\hat{M})$ values. *α* represents the conditional probability of a nucleosome staying in 1 (modified state) : *P*(*m*_*i*_ = 1|*m*_*i*−1_ = 1). *β* represents the conditional probability of a nucleosome staying in 0 (unmodified state) : *P*(*m*_*i*_ = 0|*m*_*i*−1_ = 0).

To do this, on a computer, we generated several mother sequences for different values of *α* and *β* parameters in the Markov model. The details of how the mother sequences were generated are provided in S2 Text. We then generated several daughter sequences, for each of the mother sequences, by flipping 1s to 0s randomly with probability 0.5; that is, *d*_*i*_ = *m*_*i*_ ⋅ *z*_*i*_, with *z*_*i*_ representing an element in an IID binary sequence generated by an unbiased coin. Each of the daughter sequences was corrected with the trellis-based SMAP decoding to generate the corresponding estimate $\hat{M}$; the error $\Delta (M,\hat{M})$ was computed (Eq (3)). The average of $\Delta \left(M,\hat{M}\right)=\bar{\Delta }$ (averaged over many realizations) for fixed pairs of *α* and *β*, is presented as a heatmap in Fig 3. The mean deviation ($\bar{\Delta }$) between mother and corrected mother-like daughter is low for very high values of *α* and *β*—a region dominated by long islands of ones (modified nucleosomes) separated by long islands of zeros (unmodified nucleosomes). There are other regions too where $\bar{\Delta }$ is relatively small—regions that appear more red in Fig 3—like regions with high *β* and low *α* and vice-versa. (also see S2 Text, S1 Fig). Overall, this result shows how well an ideal computing machine that employs state of the art information theory inspired algorithms can recover the original mother sequence. The remaining question is, can a real enzyme do as good as this computing algorithm?

### Threshold-*k* model: Enzymes filling unmodified islands of size at most *k* maintain chromatin fidelity

Biological enzymes being equipped to do complex SMAP computations like trellis decoding by themselves is arguably hypothetical. Nevertheless, we show that in certain biologically relevant parameter regimes, the decoding rule can be simple enough for enzymes to potentially execute. Among the known histone modification patterns, it is common to have regions densely filled by a certain modification (e.g., H3K27me3), and other regions where the modification is totally absent. Since *α* defines the probability of finding a modified nucleosome followed by another modified nucleosome, and *β* defines the probability of finding an unmodified nucleosome followed by another unmodified nucleosome, regions densely filled with a given modification would correspond to higher value of *α*; similarly, regions where the modification is totally absent would correspond to higher values of *β* in our Markov model (see Fig 1B; also see S2 Text and S2 Fig). Below we show that in this regime, the SMAP algorithm simplifies to tasks that the enzymes may easily carry out.

Consider an island of *k* unmodified nucleosomes in the daughter chromatin, giving the pattern (1, 0_*k*_, 1). From the trellis diagram (Fig 2), it can be shown that, if ${\left(\frac{\alpha }{2}\right)}^{k+1}>\frac{1}{2}\left(1-\alpha \right)\beta^{k-1}\left(1-\beta \right)$, the probability of having the all-one path (1, 1_*k*_, 1) at the mother is greater than that of (1, 0_*k*_, 1). In other words, when the function $g_{k}\left(\alpha ,\beta \right)={\left(\frac{\alpha }{2}\right)}^{k+1}-\frac{1}{2}\left(1-\alpha \right)\beta^{k-1}\left(1-\beta \right)$ is positive, an all-ones path is preferred over a run of *k* zeros by SMAP decoding. We can characterize the values of *α* and *β* for which the above condition holds true. The expression *g*_*k*_(*α*, *β*) = 0 is easy to solve if we take *k* to be a real value, this yields the root k* as:

$$\begin{array}{c} k^{*}=\frac{\text{log}(\frac{\left(1-\alpha )(1-\beta \right)}{\left(\alpha^{2}/2\right)})}{\text{log}(\alpha /2\beta )}+1. \end{array}$$ (6)

Notice that the solution for k* is unique when 0 < *α* < 1 and 0 < *β* < 1. Since $g_{1}\left(\alpha ,\beta \right)=\frac{1}{4}\alpha^{2}-\frac{1}{2}\left(1-\alpha \right)\left(1-\beta \right)$, the condition *g*_1_(*α*, *β*)>0 implies that the sequence (1, 1, 1) is preferred over (1, 0, 1). In addition, this condition will also imply that the numerator of Eq (6) is positive. The uniqueness of k* will now have the following implications:

On a unit square region of parameters (*α*, *β*) ∈ (0, 1) × (0, 1),

- When *α* < 2*β* and *g*_1_(*α*, *β*) > 0, one gets k* ≥ 1; we find that *g*_*k*_(*α*, *β*) > 0 for all positive integers *k* ≤ k* in Eq (6). This suggests that the SMAP algorithm will replace (1, 0_*k*_, 1) with (1, 1_*k*_, 1), if and only if *k* ≤ k*.
- When *α* > 2*β* and *g*_1_(*α*, *β*) > 0, we find that *g*_*k*_(*α*, *β*) > 0 for any positive integer *k*; hence the SMAP algorithm will replace (1, 0_*k*_, 1) with (1, 1_*k*_, 1), for any value of *k*.

Notice that when every path of at most k* zeros between two ones has less path metric than the corresponding all ones path, clearly any possible path other than all ones cannot have the maximum SMAP metric, while decoding sequences of length less than k*.

**Fig 4:**
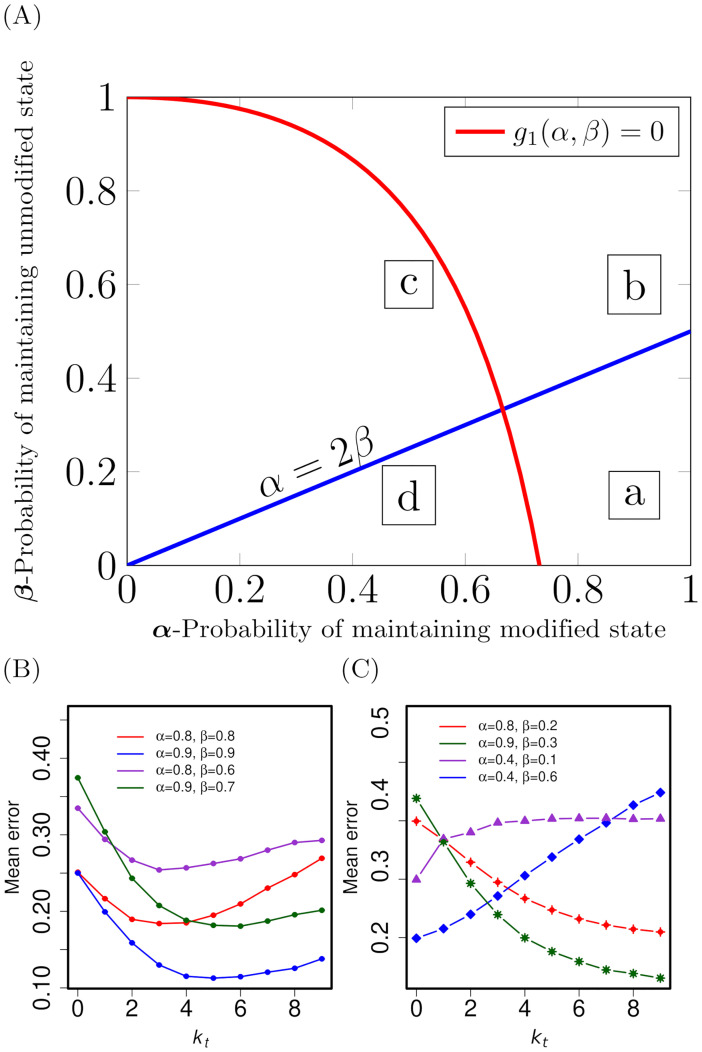
Behavior of our algorithms across the parameter spaces (A) Four (*a*, *b*, *c*, *d*) regions in (*α*, *β*) parameter space. The curves shown are *g*_1_(*α*, *β*) = 0, and *α* − 2*β* = 0. In region *a* (*g*_1_ > 0, *α* > 2*β*), the SMAP will replace every 0 with 1. In the region *b* (*g*_1_ > 0, *α* < 2*β*), enzymes can implement threshold-*k* filling (see text). This parameter regime is realistic, biologically. Region *c* has small values of *α* and relatively higher values of *β*. As per the SMAP algorithm, this region will not be filled with 1s. In region *d*, where *α* is very high compared to *β*, there is intermediate filling of 1s in the daughter sequence. (B) The mean error ($\bar{\Delta }$) when we fill all islands of 0s having size at most *k*_*t*_. All these curves have (*α*, *β*) values in region *b*, and $\bar{\Delta }$ is non-monotonic having a finite optimum *k*_*t*_ = k*. (C) $\bar{\Delta }$ for parameter values in region *a* (red and green curves) are monotonically decreasing suggesting that the optimal *k*_*t*_ is unbounded; hence the least error would be when all 0s are replaced with 1s. In regimes *c*, *d* (blue and violet curves) the mean error is minimal when nothing is filled suggesting that threshold-*k* filling is not suitable here. The standard errors here are smaller than the size of the points.

The above analysis based on trellis decoding suggests two simple ways for enzymes to work. Enzymes of Type-*I* would simply modify all unmodified nucleosomes (0s) between two modified nucleosomes (1s). Such enzymes may be preferred when the modification pattern can be modeled by parameters *α* and *β* that corresponds to region *a* in Fig 4A; notice that this has large *α* and small *β*. An enzyme of Type-*II* would fill an unmodified nucleosome (0), if and only if it falls in the region of unmodified region of length atmost ≤ k*. That is, replace (1, 0_*k*_, 1) by (1, 1_*k*_, 1), if the island size is *k* ≤ k*. Thus long islands of 0*s* are left unfilled. We call this a **threshold-*k* filling model**, which becomes active in region *b* of Fig 4A. Notice that when both *α* and *β* are close to 1, the modification is expected to have long domains (islands) with its presence, followed by islands with no modification. Biologically, this is a realistic regime for many modifications where enzymes can do threshold-*k* filling.

We tested the threshold-*k* filling model on a computer using the following procedure. For various values of *α* and *β*, we generated several mother sequences using the Markov model. The daughter sequences were generated by simulating the replication process, by randomly flipping the non-zero values using independent realizations of an unbiased coin. (See Model and Methods and S2 Text). Each daughter sequence was corrected using the threshold-*k* filling algorithm—that is, we filled all islands of 0s, having size *k* ≤ *k*_*t*_, by 1s; here *k*_*t*_ is taken as a variable. Corresponding mean error ($\bar{\Delta }$)—bit by bit difference between mother and daughter sequences—averaged over many realizations, was computed for a given (*α*, *β*). In Fig 4B, all the curves correspond to (*α*, *β*) in regime *b* of Fig 4A (*g*_1_ > 0 and *α* < 2*β*). In this interesting regime, the mean error has a non-monotonic behaviour with a minimum at a particular value of *k*_*t*_ = k*, where k* is predicted by Eq (6). Note that k* values are around 3 to 6: enzyme of Type-*II* can fill small unmodified islands having 3 to 6 zeros, and leave much longer unmodified islands unfilled.

In Fig 4C, the two monotonically decreasing curves belong to the parameter regime *a*, that is, (*g*_1_ > 0, *α* > 2*β*). The mean error is decreasing as we increase *k*_*t*_, suggesting that there is no finite k*. If the modification patterns were in this regime, the corresponding enzymes should attempt to fill every unmodified region, however small or big that may be. The other two curves in Fig 4C correspond to regimes *c* and *d* in Fig 4A. For both the curves, the mean error is increasing, suggesting that the threshold-*k* filling algorithm is not suitable in these parameter regimes.

### Threshold-*k* filling model can obtain inheritance patterns similar to what is observed experimentally

To examine the biological relevance of the findings leading to a threshold-*k* filling model, we took publicly available experimental histone modification data, and compared with our simulation results. The inheritance of modification H3K27me3 has been systematically studied recently [28] by measuring modification occupancy before and after DNA replication. Since the experimental data is population-averaged, we used a simple randomized discretization algorithm to generate many binary sequences of the available data (see S3 Text and S3 Fig).

**Fig 5:**
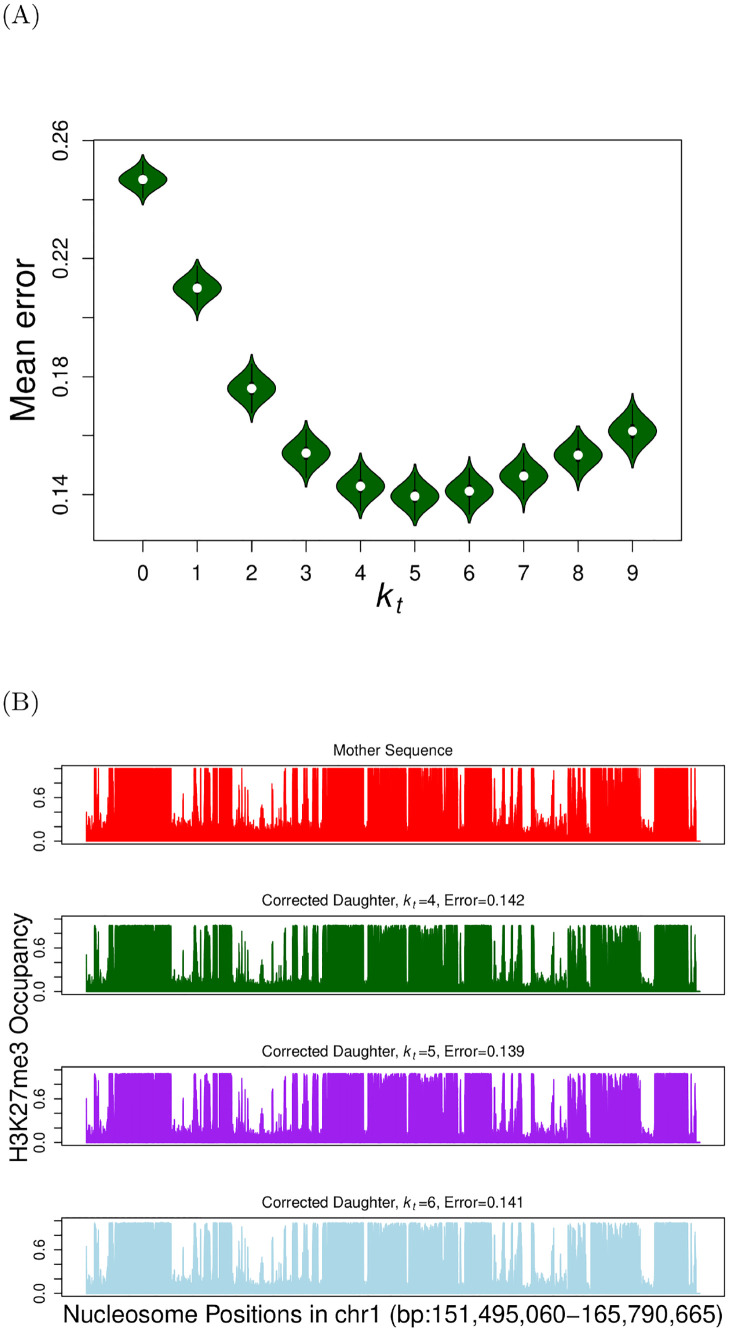
Error Correction in H3K27me3 data (A) Mean deviation ($\bar{\Delta }$) between corrected daughters and corresponding mothers, where the experimental population-averaged parental data for H3K27me3 is from [28] (see database GEO: GSE110354). Error correction was performed using the threshold-*k* filling algorithm for different *k*_*t*_ values. (B) The population averaged histone modification occupancy for H3K27me3 is plotted for mother sequence (top), and corrected daughter sequences corresponding to different values of *k*_*t*_.

This discretized binary sequence is the mother sequence *m*_*i*_. From the mother sequence, a daughter sequence ($D=\{d_{i}\}$) is created by converting each nucleosome to an unmodified state with a probability 0.5. This is equivalent to doing the mathematical operation *d*_*i*_ = *m*_*i*_ ⋅ *z*_*i*_ as mentioned earlier. The daughter sequence **D** was corrected to a mother-like modification sequence $\hat{M}$ using the threshold-k algorithm for different *k* = *k*_*t*_. This was repeated several times (100 **M** sequences, and 100 **D** for each **M**), and the mean error ($\bar{\Delta }$) is plotted as a function of *k*_*t*_ in Fig 5A. The results show that when *k*_*t*_ = 5 the mean error in the corrected H3K27me3 pattern is minimized. Note that this is very similar to the curves in Fig 4B for large values of *α* and *β*. We independently verified that the parameters corresponding to the original mother sequence is *α* ≈ 0.81 and *β* ≈ 0.815 (see S3 Text) implying that a biologically relevant modification falls in parameter regime *b*. In S4 Fig, we show the discretized mother sequences, daughter and corrected daughter sequences for a different modification (H3K4me3) whose original data was obtained from the same study [28].

In Fig 5B we plot the population-averaged modification pattern for different values *k*_*t*_. Note that islands of high and low modification occupancy regions are present in both the mother as well as the corrected daughter sequences. This indicates that our threshold *k*-filling algorithm can reproduce biologically relevant data. Thus, our information theory-inspired algorithm predicts that there might be enzymes that simply fill short segments (4 or 5 nucleosomes) of unmodified regions, but leave the longer unmodified regions (> 5 nucleosomes) unfilled. This helps in maintaining the fidelity during epigenetic inheritance. The two interesting biological questions in this context, namely (i) what are the plausible biological processes/mechanisms that could facilitate such a threshold filling process and (ii) how enzymes know what is the optimal threshold for filling, are discussed further in the Discussion section.

### Spatially distinct antagonistic modifications

The threshold-*k* filling model can be naturally extended to study two (or multiple) modifications that are antagonistic, spatially distinct (the same nucleosome will not have both the modifications simultaneously), and to be acted upon by very different enzymes. Specifically, we consider a case of a long stretch of repressive modification followed by a long stretch of activating modification in distinct neighboring regions of the chromatin. In the context of the bivalent/bistable state of the chromatin discussed in [41], it has to be noted that the antagonistic model we consider is largely not bivalent. As per our model, for an enzyme-1 responsible for modification-1, the nucleosomes having the second modification are not “visible” and would appear as a long stretch of 0s. For enzyme-2, similarly the modification-1 nucleosomes appear as a long stretch of 0s.

**Fig 6:**
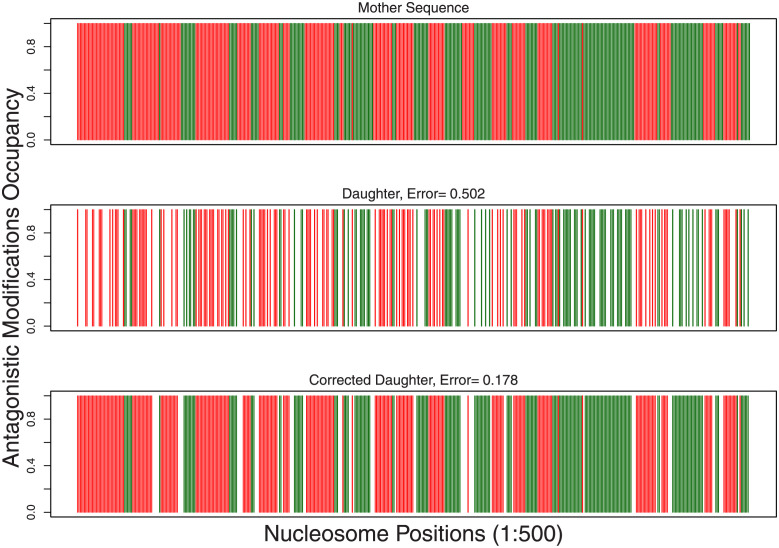
Simulating antagonistic modifications: Typical realizations of the modification patterns from the study of two spatially distinct/antagonistic modifications, spread over 500 nucleosomes. The red and green regions represent the modifications 1 and 2, respectively, and the white regions represent absence of both modifications (value 0). Corrections were performed using the threshold-*k* algorithm with an optimum *k*_*t*_ = 6.

We generated a sequence having two spatially distinct modifications. Since very high values of *α* and *β* are known to produce long regions of 1s alternating with long regions of 0s, we used this approach to generate mother sequences with long stretches of two distinct modifications—we assign them values 1 and 2 indicating the presence of modification-1 or modification-2. These are represented by red and green colors in Fig 6. Taking this as the mother sequence, daughter sequences were then obtained by simulating the replication procedure of randomly flipping the non-zero values to zero using independent realizations of an unbiased coin. Note that both 1 and 2 here are flipped to 0 indicating the fact that freshly assembled nucleosomes will have neither of these modifications. One realization of the resulting daughter sequence is shown in Fig 6 (middle panel). Then the following version of the threshold-*k* algorithm was applied to each enzyme separately. Whenever there is an island of size *k* ≤ *k*_*t*_ between two nucleosomes having modification-1, the region was filled with modification-1, similarly for modification-2. Anything else was left unfilled. This gave us results as shown in Fig 6. In Fig 6, it may be observed that while a typical daughter sequence has an error ∼ 0.5, the corrected daughter had an error less than 0.2. These are occupancies from a typical realization (not averaged over the population) and hence the values are either 0 or 1.

It has to be noted that this is only a preliminary extension of our single modification inheritance model to a pair of antagonistic modifications. In reality, the process of filling, in case of multiple modifications may involve intermediate steps by several enzymes. This could be explored potentially as part of a future work.

## Discussion

In this work, we proposed that the problem of the daughter chromatin retrieving histone modification patterns, to achieve a mother-like chromatin state, can be mapped to a communication theory problem of receiving noisy signal and correcting it to retrieve the original signal. Using ideas from information theory, we argued that if enzymes were ideal computing machines, they could mimic a MAP decoding algorithm to get back a mother-like sequence. We showed how well this algorithm would reconstruct the mother—the error can be as low as 5% in certain parameter regimes. However, the question was whether realistic enzymes can practically do SMAP decoding. We showed that in a biologically relevant parameter regime, MAP decoding algorithm is equivalent to a threshold-*k* filling algorithm. That is, the enzymes could simply insert modifications in *k*-sized or shorter unmodified stretches (0s). The fact that a detailed theory simplifies to a process that is potentially executable by enzymes makes this result attractive. Below, we discuss some aspects of our model, predictions and future directions, in the context of the existing literature.

**Our results in the context of existing models**: Currently, there are different categories of models that study various aspects of inheritance. First category belongs to computational models that study regions that are bistable. Work from Sneppen, Dodd, Ringrose, Howard and others have been discussing aspects of bistability [40, 41, 44]. This approach, in particular, is useful to explain many regions on stem cell chromatin, bivalent regions, specific regions like mating type locus in yeast and so on. In this model, the region of interest switches between two states—e.g., active and inactive—as a function of time; this would also imply that, at any given time, there are two populations of cells in a large ensemble. In the work of Sneppen and co-workers, “state” is quantified by finding the mean modification (over all modified/unmodified nucleosomes) in a given region and is independent of the parental chromatin state as long as there is a sufficient number of modified nucleosomes. They do not study the precise spatial pattern in detail. In contrast, there are regions that are stably maintained in a certain modified state and have specific pattern of histone modifications. This leads to a second category of models: e.g., models by Hodges and Crabtree, and others [45, 46] that study the spreading and maintaining of histone modifications as stable modified patterns over a finite length region in the steady state. This is achieved by introducing a nucleation site (which could be non-epigenetic like a DNA sequence-dependent location) that acts like a source for modifications/modifying enzymes. While these models explain maintenance of modifications, they require a nucleation site and have a decaying pattern from the nucleation site.

The above two categories of models start with certain physics-based kinetic moves for enzymes and ask what the steady state is. We take a different approach—an information/communication theory based approach—and ask the following reverse question : What must enzymes do to achieve a high-fidelity inheritance? We find that threshold-*k* filling is the answer that information/communication theory provides. While we do not have kinetics in our model, we show (in S5(A) Fig) that if the parental population of cells have two stable states (bistable), the inherited population will also have two stable states. This can be considered as the ensemble interpretation of bistable states [40, 41, 44]. We also show (S5(B) Fig) that if the parental population has a stable modified state, it will also be stably inherited like in the case discussed in [45]. See S3 Text.

**Physical/Biological Relevance of the Model**: We modeled the mother chromatin as a first order Markov process. This is a reasonable model as there are just two parameters, *α* and *β*, which are explicitly related to the experimentally measurable properties of the modification patterns such as the mean contiguous length of modified and unmodified regions (see S2 Text). By varying the threshold *k*_*t*_, as shown in Figs 4B and 5A, we can determine the optimal configurations and obtain insights about how enzymes might work without a-priori knowledge of the statistical parameters. Note that even in the large *α* regime (region *a* in Fig 4A), if enzymes settle for threshold filling with a finite *k*_*t*_ (e.g., 5 or 6), it becomes a pragmatic modification correction solution as the resulting error is relatively low (see red and green curves in Fig 4B).

It has to be noted that our model picks configurations (sequences) that maximize certain conditional probabilities, analogous to the minimum energy (or “equilibrium”) solution in statistical physics [60, 61]. The model does not involve precise kinetic moves. There could be multiple different kinetic events leading to the same static configuration. Hence, the predictions of our model may be interpreted in terms of various known kinetic events in the field. For example, the determination of critical threshold *k*_*t*_ as well as the decision to fill the gaps would involve local read-write mechanisms as well as feedback loops. The presence of a modification (1) inducing more modifications nearby, is an example of positive feedback. The decision to leave long gaps unfilled also may involve certain feedback mechanisms.

While enzymes fill the unmodified nucleosomes based on modified inherited nucleosomes, there is a natural question: how far away can the modification spread, from a modified nucleosome. Our model gives an answer to this question: it can spread upto *k* sites from a modified nucleosome. Even starting with nearest neighbour Markov model, there is a correlation length emerging that is *k* sites long. Our threshold-*k* model provides a natural length-scale (of length *k*_*t*_) for inter-nucleosomal interactions. Moreover, our model has the advantage that it reproduces a spatial pattern similar to that of the parent.

**How plausible is threshold-*k* filling in chromatin?**: What might be the biological processes that keep a group of nucleosomes in a modified state or unmodified state is an interesting question. A region can be kept unfilled as a combined effect of modification and de-modification (de-methylation, de-acetylation etc.) events. When the rate of de-modification in a region is much higher than the modification rate, the region can remain unfilled. Physically, this could happen in several ways. Enzymes could cooperatively act on a small region. For example, interesting recent experimental studies [65] suggest that enzymes that act on histone modifications can phase separate to form a droplet around a group of nucleosomes, and may collectively modify/demodify all the nucleosomes together. It has also been shown that chromatin itself can form nano-domains having size of a few nucleosomes paving way for collectively maintaining a certain modification state in these groups of nucleosomes [66–68]. The potential enzymes/complexes that could do these activities could be JmjC domain proteins, UTX, NuRD, Fbxl10 and JARID1A [41, 69, 70]. In case of H3K27me3 in particular, experimental evidence supports that EZH2 and H2AK119Ub also contribute to the modification of the neighboring nucleosomes [26, 71]. Approaching this question from a different angle, currently, it is well-known that there exist certain correlations and anti-correlations between modifications [14]. There are biological mechanisms which are known to maintain these correlated states between modifications [38]. Hence, the mechanism that may cause the absence of a modification could also cause the presence of an opposite modification as we have discussed in the antagonistic modifications section. For example, recent experiments from [47, 72] suggest that there is mutual antagonism between H3K27 and H3K36 methylation. This antagonism could also be one of the reasons for a long set of nucleosomes in which one modification is absent.

How enzymes know what is the optimal threshold for filling, is another interesting question. In recent experiments, it was shown that a critical density of H3K9me3 was required for stable transgenerational inheritance of heterochromatin [31]. It has to be noted that our proposed model in which a critical threshold of parental modifications is required for filling the gaps of unmodified nucleosomes, is strengthened with this result. There are different families of enzymes, and each may have evolved differently. Some enzymes may be naturally adept at filling short regions, i.e. optimal threshold value (k*) could be hard-wired into such enzymes, via evolution. Another possibility is that other phenomena like local looping, phase separation etc. decide the threshold k*, by bringing unfilled nucleosomes together [73, 74]. These need to be understood in more detail in the future. As we showed, the information theory ideas suggest that the threshold-*k* filling algorithm emerges as a natural primary solution. However, further reconstruction may involve secondary mechanisms like boundary determination (e.g. CTCF [75]), synergistic modification between correlated modifications, and DNA methylation maintenance over the course of the cell-cycle [25, 38].

**Inheritance over many generations**: Another interesting aspect is the fidelity of inheritance over many generations. Error propagation across generations will depend on how well reconstruction happens in the very first generation. In the Figs 3 and 4, we have shown that the error (if we compute it as bit error rate—BER) is a non-zero number. Any non-zero error will propagate over generations. None of the published models so far, to the best of our knowledge, compare the mother and daughter modifications bit by bit and compute the error, they rather compute the mean modifications (modifications averaged over a long stretch) or the population-averaged pattern. In this work, we consider both the mean modification/mean pattern and bit by bit error (see S3 Text).

As pointed out above, we argue that threshold-*k* filling algorithm emerges as a natural primary solution (immediately post-replication) based on information theoretic principles. However, in addition to this, the reconstruction may involve secondary mechanisms like boundary determination via binding of proteins like CTCF, formation of nucleosome free regions [20, 25, 76–79] and so on. In reality, all these mechanisms, together, will contribute to reconstruction and propagation of epigenetic information with minimum error.

Even in the absence of secondary mechanisms, we show that our model approximately preserves the pattern of modifications across a few generations (S6 Fig). We have simulated the case of repeated replication over 3 generations for a biologically realistic regime of *α* and *β* = 0.9 each. When the deviation (BER) is computed as against the original set of mother sequences with the above statistical parameters, we observe that deviation is reasonably low for up to 5 generations for *k*_*t*_ = 6 (see S3 Text and S6 Fig). We also computed the block error—error averaged over blocks of neighboring nucleosomes—and plotted the results in S6(A) Fig. From the figure, one can observe that the error decreases with increasing block size. The BER and the mean block error remain very small across generations (see S3 Text). We also show in S6(B) Fig that the mean modification pattern is maintained across generations, using our algorithm on the H3K27me3 data. The details are provided in S3 Text.

Finally, it would also be of great interest to study how the polymer nature of the chromatin would work in tune with the results from information theory. It has to be noted that the epigenetic code might involve an interplay between the one-dimensional histone codes and polymer dynamics of the chromatin [80, 81]. The fact that we have two 1*s* at the boundaries suggests some potential role of looping or micro phase separation in far-away regions coming together. Electrostatic interactions among nucleosomes could also play an important role here [82–87]. For example, the charge distribution of a bunch of de novo nucleosomes can lead to a loose clustering of *k* nucleosomes, thereby facilitating the threshold-*k* filling. Our own earlier studies [68] hint to us that small patches of unmodified nucleosomes (newly inserted nucleosomes) may lead to small clusters, influencing the kinetics of the modification process itself. Additionally it might be interesting to analyse how the inheritance is affected in cases where there may be unequal distribution of the parental nucleosomes between the leading and lagging strands [21]. These are questions that await future studies.

## Supporting information

S1 FigAnalysis of Mean Error after correction.In (A) and (C), we plot the mean error after correction ($\bar{\Delta }$) using the SMAP algorithm, as a function of *α* for different values of *β*. In (B) and (D), we plot the mean error after correction as a function of *β* for different values of *α*. The error bars representing SEM are are smaller than the size of the points in the plots. *α* represents the conditional probability of a nucleosome staying in 1 (modified state): *P*(*m*_*i*_ = 1|*m*_*i*−1_ = 1). *β* represents the conditional probability of a nucleosome staying in 0 (unmodified state): *P*(*m*_*i*_ = 0|*m*_*i*−1_ = 0). The error is averaged over a simulation of 300 mother sequences each of which has 200 daughter sequences (a total of 60000 cases). (E) represents the variation of error after correction for different lengths of the sequence, for *α* and *β* values of 0.9 each. (F) and (G) represent normalized mean error after correction obtained varying against *k*_*t*_—these plots are similar to the Fig 4B and 4C in the manuscript but are normalized with respect to the optimum error.(EPS)

S2 Fig Simulated and Binarized sequences. Binarized sequences (top panel) for H3K27me3 and H3K4me3 from HeLa cells (discretized using the algorithm mentioned in S3 Text) and simulated sequences (bottom panel) corresponding to the same *α* and *β*. The mean length of the modified and unmodified regions (runs) are distributed in a statistically similar manner in both the cases, suggesting that Markov model is a reasonable starting point.
(EPS)

S3 Fig Discretization Algorithm. The algorithm used to discretize the population-averaged parental modification data (*X*) from [28] into a binary realization—equivalent of a single cell representation indicating the presence (1) or absence (0) of the modification. In our case, *X* is H3K27me3 or H3K4me3 data obtained from [28].
(EPS)

S4 Fig H3K4me3 Experimental Verification. Mother, Daughter, and the Corrected sequences for *k*_*t*_ = 4, 5, 6 for the H3K4me3 values in the HeLa cells from a region of the Chr19 of the human genome. The data was obtained from [28] and is averaged over 200 binary realizations of the mother, each having 100 daughters.
(EPS)

S5 Fig Simulation in the context of existing models. The histograms of the mother and corrected daughter sequences in the case where the mother sequences exhibit a bistable population of cells (i.e), roughly half of the cells have high modification levels whereas the remaining half of the cells have low modification levels. The replicated daughter cells after correction by our algorithm also show a similar bistable distribution. In (B), the plots of the mean mother and corrected daughter sequences for the case of a mother having a pattern similar to the one observed in [45] are shown. After correction by our algorithm, the daughter sequences obtain a pattern very similar to what is observed in the mother.
(EPS)

S6 Fig Error across generations. (A)—The plot of the mean block error between the original mother sequence and the corrected daughter sequences across subsequent generations for *α* and *β* values of 0.9 each. With the optimum value of *k*_*t*_ for this range of *α* and *β* (*k*_*t*_ = 6), the mean error increases minimally over subsequent replications. With increasing block size, the mean error decreases monotonically. When the block size is 1, the error is the Bit Error Rate (BER). In (B), the plots of the mean mother and corrected daughter sequences for different generations are shown. The data is obtained from [28] and is the population-averaged binarized sequences of H3K27me3 ChIP-seq data in the Chr1 region.
(EPS)

S1 Text SMAP Algorithm, Markov Process, Trellis Diagram. (PDF)

S2 Text Computation of Statistical Properties. (PDF)

S3 Text Discretization Algorithm, Experimental Data Simulation, Error across Generations. (PDF)
