# Supplementary material for: High fidelity epigenetic inheritance: Information theoretic model predicts threshold filling of histone modifications post replication
Source: PLoS Comput Biol. 2022 Feb 17;18(2):e1009861. doi: 10.1371/journal.pcbi.1009861 (PMC8903295; doi:10.1371/journal.pcbi.1009861)
Supplement: S1 Text — (PDF) [file pcbi.1009861.s007.pdf]

Supplementary Text S1

High fidelity epigenetic inheritance: Information theoretic model predicts threshold filling of histone modifications post replication

Maximum À-posteriori Probability Decoding

As illustrated in Fig 1C of the manuscript, the vector $\mathbf{M}$ represents the binary mother sequence, and each non-zero value of this sequence is independently flipped with probability half to obtain the daughter sequence $\mathbf{D}$. This daughter sequence represents one of the two possible chromatins post replication. The flipping operation is equivalent to a logical AND between the independent sequences $\mathbf{M}$ and $\mathbf{Z}$, where the $\mathbf{Z}$ is independently and identically distributed (IID) according to an unbiased binary random variable. The goal now is to restore a mother-like pattern $\hat{\mathbf{M}}$ from $\mathbf{D}$. Recall the notation $m_i^j = m_i, m_{i+1}, \ldots, m_j$ introduced in the manuscript. Using this, let m_1^N represent a realization of the mother sequence $\mathbf{M}$ of length N.

We use the MAP (Maximum À-posteriori Probability) rule to reconstruct a mother like sequence from the daughter. The MAP rule [1] suggests to choose the sequence $\hat{\mathbf{M}}$ which maximizes the à-posteriori probability $\mathbb{P}(\hat{\mathbf{M}}|\mathbf{D})$. Thus, the decoder chooses $\hat{\mathbf{M}}$ such that

$$\begin{aligned}\hat{\mathbf{M}} = \underset{\mathbf{M}}{\operatorname{argmax}}\ \mathbb{P}(\mathbf{M}|\mathbf{D}) &= \underset{\mathbf{M}}{\operatorname{argmax}} \left(\frac{\mathbb{P}(\mathbf{M})\mathbb{P}(\mathbf{D}|\mathbf{M})}{\mathbb{P}(\mathbf{D})}\right) \\ &= \underset{\mathbf{M}}{\operatorname{argmax}} \big(\mathbb{P}(\mathbf{M})\mathbb{P}(\mathbf{D}|\mathbf{M})\big).\end{aligned} \tag{S1}$$

The first step in Eq. S1 follows from the Bayes theorem. The second step uses the fact that $\mathbb{P}(\mathbf{D})$ is effectively a scaling factor, and the argument which maximizes is it unchanged by removing any common scaling.

The quantity $\mathbb{P}(\mathbf{M})$ in Eq. (S1) is governed by Eq. (1) in the manuscript.

$$\mathbb{P}\big(\mathbf{M} = m_1^N\big) = \mathbb{P}(m_1)\prod_{i=2}^{N}\mathbb{P}(m_i|m_{i-1}). \tag{S2}$$

Taking $m_0 = \emptyset$ (empty set), we can write

$$\mathbb{P}\big(\mathbf{M} = m_1^N\big) = \prod_{i=1}^{N}\mathbb{P}(m_i|m_{i-1}). \tag{S3}$$

To prove Eq. (2) in the manuscript,

$$\begin{aligned}\mathbb{P}(\mathbf{D} = d_1^N|M = m_1^N) &= \mathbb{P}(d_1, d_2, \ldots, d_N\ |m_1^N) \\ &= \mathbb{P}(d_1 \mid m_1^N)\mathbb{P}(d_2 \mid m_1^N, d_1)\ldots\mathbb{P}(d_N \mid m_1^N, d_1^{N-1}) \\ &= \mathbb{P}(d_1|m_1)\mathbb{P}(d_2|m_2)\ldots\mathbb{P}(d_N|m_N) \\ &= \Pi_{i=1}^N\mathbb{P}(d_i|m_i).\end{aligned} \tag{S4}$$

The second step in Eq. (S4) follows from Bayes rule. Since $d_i = m_i \cdot .z_i$, and the realization z_i being independent of $(z_1^{i-1}, z_{i+1}^N, m_1^N)$ by our IID assumption on flipping, we have $\mathbb{P}(d_i|m_1^N, d_1^{i-1}) = \mathbb{P}(d_i|m_i)$. Notice that this corresponds to the memoryless nature of the flipping operation.

Thus Eq. (5) of the manuscript follows from equations Eq. (S3) and Eq. (S4).

Markov Process, Trellis Diagram and Branch Probabilities

Since a modification is randomly and independently copied to one of the daughters, we take the definition of our modification flipping operation, we obtain

$$\mathbb{P}\big(d_i = 0 \mid m_i = 0\big) = 1 \tag{S5}$$

$$\mathbb{P}\big(d_i = 1 \mid m_i = 1\big) = \mathbb{P}\big(d_i = 0|m_i = 1\big) = \frac{1}{2}. \tag{S6}$$

The ideas behind trellis decoding is best illustrated with a generic example. Given a daughter sequence d_1^N, consider two possible mother sequences a_1^N and b_1^N. The MAP rule will prefer sequence a_1^N over b_1^N if the joint probability law obeys $\mathbb{P}(a_1^N, d_1^N) > \mathbb{P}(b_1^N, d_1^N)$. The trellis diagram, depicted in Fig 2 of the manuscript, is an effective way to compute and compare these joint probabilities. We can limit our considerations to sequences a_1^N, b_1^N and d_1^N which start and end with the value 1, since $d_1^N = (1, 0_{N-2}, 1)$ is given as the observed sequence. In the expressions below, joint probability terms of the form $\mathbb{P}(x, y|w)$ will have the first variable x representing the mother and the second variable y representing the daughter, at a particular nucleosome index.

$$\begin{aligned}\mathbb{P}(a_1^N, d_1^N) &= \mathbb{P}(1,1)\left(\prod_{i=2}^{N-1} \mathbb{P}(a_i, d_i = 0|a_{i-1})\right)\mathbb{P}(a_N, d_N = 1|a_{N-1}) \\ &= \mathbb{P}(1,1)\left(\prod_{i=2}^{N-1} \mathbb{P}(a_i|a_{i-1})\mathbb{P}(d_i = 0|a_i)\right)\mathbb{P}(a_N|a_{N-1})\mathbb{P}(d_N = 1|a_N).\end{aligned} \tag{S7}$$

Similarly,

$$\mathbb{P}(b_1^N, d_1^N) = \mathbb{P}(1,1)\left(\prod_{i=2}^{N-1} \mathbb{P}(b_i|b_{i-1})\mathbb{P}(d_i = 0|b_i)\right)\mathbb{P}(b_N|b_{N-1})\mathbb{P}(d_N = 1|b_N). \tag{S8}$$

In the both the above equations we used the fact that $a_1 = b_1 = d_1 = d_N = 1$. Notice that, while comparing (S7) and (S8), we can ignore the initial common scaling factor $\mathbb{P}(1,1)$. The trellis diagram enables finding the remaining product by assigning branch metrics to the $N-1$ possible transitions of each path. In particular, the ith transition corresponding to the sequence a_1^N will have an associated branch metric $\mathbb{P}(m_{i+1}|m_i)\mathbb{P}(d_{i+1}|m_{i+1})$ in the trellis diagram. From our Markov model, the probabilities $\mathbb{P}(m_i, d_i|m_{i-1})$ take the form

$$\mathbb{P}(0,0|0) = \beta \tag{S9}$$

$$\mathbb{P}(0,0|1) = 1-\alpha \tag{S10}$$

$$\mathbb{P}(1,0|0) = \mathbb{P}(1,1|0) = \frac{1-\beta}{2} \tag{S11}$$

$$\mathbb{P}(1,0|1) = \mathbb{P}(1,1|1) = \frac{\alpha}{2} \tag{S12}$$

$$\mathbb{P}(0,1|0) = \mathbb{P}(0,1|1) = 0. \tag{S13}$$

Let us now illustrate the trellis computations for a specific example. Take $N = 4$, and $a_1^4 = (1,1,1,1)$ and $b_1^4 = (1,0,0,1)$. The observed daughter sequence is given as $(d_1, d_2, d_3, d_4) = (1,0,0,1)$.

$$\mathbb{P}(a_1^4, d_1^4) = \mathbb{P}(1,1)\mathbb{P}(1,0|1)\mathbb{P}(1,0|1)\mathbb{P}(1,1|1) \tag{S14}$$

$$= \mathbb{P}(1,1)\frac{\alpha}{2}\frac{\alpha}{2}\frac{\alpha}{2} \tag{S15}$$

$$= \mathbb{P}(1,1)\,\frac{\alpha^3}{8}. \tag{S16}$$

$$\mathbb{P}(b_1^4, d_1^4) = \mathbb{P}(1,1)\mathbb{P}(0,0|1)\mathbb{P}(0,0|0)\mathbb{P}(1,1|0) \tag{S17}$$

$$= \mathbb{P}(1,1)\left((1-\alpha)\,\beta\,\frac{1-\beta}{2}\right) \tag{S18}$$

$$= \mathbb{P}(1,1)\,\frac{(1-\alpha)\beta(1-\beta)}{2}. \tag{S19}$$

Trellis decoding will compute $\frac{\alpha^3}{8}$ as the metric for the path a_1^4, whereas $\frac{1}{2}(1-\alpha)\beta(1-\beta)$ will be the metric of the path b_1^4. As another example, from Fig 2 of the manuscript, the metric for the path $(1,0,0,0,0,0,1)$ can be easily computed by traversing through the corresponding path and reading out the product of branch metrics encountered, leading to a final metric $(1-\alpha)\beta^5\frac{1}{2}(1-\beta)$.

SMAP Decoding Proposition Proof

Proposition 1 *Let i, j be two positions where the daughter sequence has ones, with $j > i$. Then, SMAP decoding will choose a sequence having $\hat{m}_i^j$ according to*

$$\hat{m}_i, \ldots, \hat{m}_j = \text{argmax}\ \mathbb{P}(\mathrm{m_i}, \ldots, \mathrm{m_j} | \mathrm{d_i} = 1, \mathrm{d_{i+1}^{j-1}}, \mathrm{d_j} = 1).$$

Notice that $\mathbb{P}(m_i, d_i | m_{i-1}) = \mathbb{P}(m_i | m_{i-1})\mathbb{P}(d_i | m_i, m_{i-1}) = \mathbb{P}(m_i | m_{i-1})\mathbb{P}(d_i | m_i)$, by Bayes rule and Eq. (2) in the manuscript. Using this in Eq. (5) of the manuscript,

$$\mathbb{P}(\mathbf{M}|\mathbf{D}) = \frac{1}{\mathbb{P}(\mathbf{D})} \prod_{l=1}^{i-1} \mathbb{P}(m_l, d_l | m_{l-1}) \mathbb{P}(m_i | m_{i-1}) \mathbb{P}(d_i | m_i) \prod_{l=i+1}^{j} \mathbb{P}(m_l, d_l | m_{l-1}) \prod_{l=j+1}^{N} \mathbb{P}(m_l, d_l | m_{l-1}). \tag{S20}$$

Given the actual value of m_j, determining the bits $\hat{m}_{j+1}^N$ can be easily seen from above as

$$\hat{m}_{j+1}^N = \text{argmax} \prod_{l=j+1}^{N} \mathbb{P}(m_l, d_l | m_{l-1}). \tag{S21}$$

Similarly, once m_i is fixed, then the bits $\hat{m}_1^{i-1}$ are determined by

$$\hat{m}_1^{i-1} = \text{argmax} \prod_{l=1}^{i-1} \mathbb{P}(m_l, d_l | m_{l-1}) \mathbb{P}(m_i | m_{i-1}). \tag{S22}$$

Observe that both Eq. (S21) and Eq. (S22) are independent of m_{i+1}^{j-1}. Given $d_i = d_j = 1$, only sequences with $m_i = m_j = 1$ are to be considered for our maximization, since $\mathbb{P}(m_i = 1 | d_i = 1) = 1$. Thus, the subsequences $\hat{m}_1^{i-1}$ and $\hat{m}_{j+1}^N$ are determined independently of $\hat{m}_{i+1}^{j-1}$, as shown above. Therefore we get

$$\hat{m}_{i+1}^{j-1} = \underset{m_i^j : m_i = m_j = 1}{\text{argmax}} \prod_{l=i+1}^{j} \mathbb{P}(m_l, d_l | m_{l-1}) \tag{S23}$$

as the output subsequence of SMAP decoding. The proof of the proposition is completed by noting that $\mathbb{P}(m_i^j | d_i^j) = \mathbb{P}(m_i^j, d_i^j)/\mathbb{P}(d_i^j)$ and $\mathbb{P}(m_i^j, d_i^j) = \prod_{l=i}^{j} \mathbb{P}(m_l, d_l | m_{l-1})$.

---

[1] Cover TM, Thomas JA. 2012 *Elements of information theory.* John Wiley & Sons.

[2] Sneppen K, Ringrose L. 2019 Theoretical analysis of Polycomb-Trithorax systems predicts that poised chromatin is bistable and not bivalent. *Nature communications* **10**, 1–18.

[3] Swygert SG, Peterson CL. 2014 Chromatin dynamics: interplay between remodeling enzymes and histone modifications. *Biochimica et Biophysica Acta (BBA)-Gene Regulatory Mechanisms* **1839**, 728–736.

[4] Lin S, Costello D. 2005 *Error Control Coding.* Pearson Education.
