# Supplementary material for: High fidelity epigenetic inheritance: Information theoretic model predicts threshold filling of histone modifications post replication
Source: PLoS Comput Biol. 2022 Feb 17;18(2):e1009861. doi: 10.1371/journal.pcbi.1009861 (PMC8903295; doi:10.1371/journal.pcbi.1009861)
Supplement: S2 Text — (PDF) [file pcbi.1009861.s008.pdf]

# Supplementary Text S2

## High fidelity epigenetic inheritance: Information theoretic model predicts threshold filling of histone modifications post replication

### Computation of Statistical Properties

In this section, we explain how the parameters $\alpha$ and $\beta$ for a given binary sequence, modeled by the Markov process described in Fig 1B of the manuscript, can be estimated. Consider a realization $\{m_1, m_2, \ldots, m_N\}$ of the binary sequence $\mathbf{M}$. We define the following variables as the number of transitions:

$$\begin{aligned} n_{11} &- \text{Number of transitions from 1 to 1} \\ n_{10} &- \text{Number of transitions from 1 to 0} \\ n_{01} &- \text{Number of transitions from 0 to 1} \\ n_{00} &- \text{Number of transitions from 0 to 0.} \end{aligned} \tag{S1}$$

While traversing through the sequence, we increment the transition variables as follows:

$$\begin{aligned} n_{11} &= n_{11} + 1; \text{ if } (m_{i-1} = 1 \text{ AND } m_i = 1); \\ n_{10} &= n_{10} + 1; \text{ if } (m_{i-1} = 1 \text{ AND } m_i = 0); \\ n_{01} &= n_{01} + 1; \text{ if } (m_{i-1} = 0 \text{ AND } m_i = 1); \\ n_{00} &= n_{00} + 1; \text{ if } (m_{i-1} = 0 \text{ AND } m_i = 0). \end{aligned} \tag{S2}$$

We then compute $\alpha$ and $\beta$ as:

$$\alpha = \frac{n_{11}}{(n_{11} + n_{10})} \tag{S3}$$

$$\beta = \frac{n_{00}}{(n_{00} + n_{01})}. \tag{S4}$$

The biological processes that keep nucleosomes in a modified state or unmodified state (leading to the parameters $\alpha$ and $\beta$) include co-operative modification/de-modification between correlated modifications, the effect of the presence of antagonistic modifications in certain nucleosomes, amongst others. When the rate of de-modification is high as compared with the rate of modification, longer runs of 0s can be observed. Some potential enzymes that perform de-modification (de-methylation, de-acetylation , de-ubiquitination) could be JmjC domain proteins and UTX , NuRD, Fbxl10 and JARID1A [1, 2].

**Performance of MAP decoding:** In communication, it is well-known that the optimal BER performance can be achieved by Bitwise MAP (BMAP) decoding, however this scheme is not only computationally more demanding, but also analytically less tractable [3]. In the biological context, it appears that SMAP decoding is a more probable candidate for cells to reconstruct the epigenetic modifications from the partial data. Notice that SMAP decoding depends on the parameters $\alpha$ and $\beta$ of the Markov chain.

### Simulation Procedure and Additional Results

We simulated the mother sequences in a computer using different values of $\alpha$ and $\beta$ for the Markov process. Each mother sequence $m_1^N$ was generated as follows: We start with the first bit as 1 ($m_1 = 1$ ) . Then, the next bit is inserted as 1 with a probability $\alpha$, or 0 with probability $1 - \alpha$. That is, if a uniform random number $r_i$ is less than $\alpha$ , then the next bit inserted as 1, else as a 0. Once there is a 0 in the sequence, the next bit is inserted as 0 with a probability $\beta$, or 1 with probability $1 - \beta$. In general, a bit ($m_i$=0) is maintained as ($m_{i+1}$=0) with probability $\beta$ while a bit ($m_i$=1) is maintained as ($m_{i+1}$=1) with probability $\alpha$.

Given a mother sequence, daughter sequences were generated by randomly flipping the non-zero values using independent realizations of an unbiased coin. 200 such daughter realizations were generated for each mother sequence considered. Each daughter sequence was corrected using the SMAP decoding rule, and compared to its mother to

obtain the mean error ($\bar{\Delta}$). The error was averaged over all the 200 daughter sequences, and the whole experiment was repeated 300 times to obtain the averaged error (averaged over a total of 60000 cases). The length of the sequence was taken as 100 nucleosomes in the simulations. The variation of the mean error across $\alpha$ and $\beta$ are plotted in S1 A-D Figs. Comparing S1 A-D Figs and Fig 3 (in the manuscript), which has the heatmap of the mean error after correction, we can identify regions of low and high error. Furthermore, the behaviour shown in the plots S1 A-D Figs is related to the phase diagram Fig 4A of the manuscript. S1E Fig represents the variation of the mean error after correction for different values of $N$ (lengths of sequences) for $\alpha$ and $\beta$ values of 0.9 each. S1F and S1G Figs represent normalized mean error after correction obtained varying against $k_t$ - these plots are similar to the Fig 4B and Fig 4C in the manuscript but are normalized with respect to the optimum error.

## Average number of 0s and 1s in the mother sequence

The steady state probabilities ($\Pi_1$ and $\Pi_0$) of states '1' and '0' in a binary sequence modeled by a Markov chain described in Fig 1B of the manuscript are given by the following equations [4]:

$$\Pi_1 = \frac{(1-\beta)}{(2-\alpha-\beta)} \tag{S5}$$

$$\Pi_0 = \frac{(1-\alpha)}{(2-\alpha-\beta)}. \tag{S6}$$

The average number of 1s and 0s in the mother sequence is given by the steady state probabilities of states '1' and '0' in Eq. (S5) and Eq. (S6), multiplied by the length of the sequence.

## Average number of intermediate 0s and 1s

Let us compute the average number of intermediate 0s between two 1s in a long sequence of the mother sequence. Based on our Markov model, the run of zeros can be considered as a geometric random variable that lists the number of 0s before the next 1 is encountered. The probability of getting a 1 after a 0 is $1-\beta$. Thus, given that there is at least one 0, the expected number of 0s between two 1s is the mean of this geometric random variable, given by

$$\text{Expected number of intermediate 0s} = \frac{1}{1-\beta}. \tag{S7}$$

In other words, this is the mean contiguous length of intermediate 0s.

Similarly, the average length of a run of 1s between two bordering 0s can be computed as

$$\text{Expected number of intermediate 1s} = \frac{1}{1-\alpha}. \tag{S8}$$

Both these quantities can be obtained from experimental data.

From Eq. S5, Eq. S6 and the values of the average number of intermediate 0s and 1s , we understand that when $\alpha$ and $\beta$ are very high, the average run of 0s (or 1s) will be high. This would result in high chances of finding a long stretch of 0s, or a long island of 1s, for large values of $\alpha$ and $\beta$. The average run lengths of 0s (unmodified nucleosomes) and 1s (modified nucleosomes) for different values of $\alpha$ and $\beta$ are provided in Table A.

[1] Sneppen K, Ringrose L. 2019 Theoretical analysis of Polycomb-Trithorax systems predicts that poised chromatin is bistable and not bivalent. *Nature communications* **10**, 1–18.
[2] Swygert SG, Peterson CL. 2014 Chromatin dynamics: interplay between remodeling enzymes and histone modifications. *Biochimica et Biophysica Acta (BBA)-Gene Regulatory Mechanisms* **1839**, 728–736.
[3] Lin S, Costello D. 2005 *Error Control Coding.* Pearson Education.
[4] Stewart WJ. 2009 *Probability, Markov chains, queues, and simulation: the mathematical basis of performance modeling.* Princeton university press.

| $\alpha$ | $\beta$ | Average runs of unmodified nucleosomes | Average runs of modified nucleosomes |
|---|---|---|---|
| 0.73 | 0.85 | 5.5 | 4.03 |
| 0.87 | 0.77 | 5.08 | 5.91 |
| 0.633 | 0.781 | 2.63 | 5.84 |
| 0.45 | 0.95 | 15.3 | 1.89 |

Table A. Average run lengths of modified and unmodified nucleosomes for different values of $\alpha$ and $\beta$.
