# Supplementary material for: High fidelity epigenetic inheritance: Information theoretic model predicts threshold filling of histone modifications post replication
Source: PLoS Comput Biol. 2022 Feb 17;18(2):e1009861. doi: 10.1371/journal.pcbi.1009861 (PMC8903295; doi:10.1371/journal.pcbi.1009861)
Supplement: S3 Text — (PDF) [file pcbi.1009861.s009.pdf]

Supplementary Text S3

High fidelity epigenetic inheritance: Information theoretic model predicts threshold filling of histone modifications post replication

Discretization algorithm

The population-averaged parental modification data obtained from the experiments in [1] were converted to binary realizations of a single-cell indicating the presence or absence of the modification in a nucleosome. The algorithm is explained in the flowchart of S3 Fig. It has to be noted that the H3 experimental data was also used in the algorithm in order to detect the presence of the nucleosomes as against finding the presence of the modification in the nucleosome. In S2 Fig, the binarized versions of the H3K27me3 and H3K4me3 data in various regions of the genome, discretized using the algorithm in S3 Fig are shown. The corresponding α and β values indicate that these regions fall under the b region of Fig 4A in the manuscript. Simulated sequences for the corresponding α and β values show the similarity in the run lengths of the 0 and 1 islands between the genomic and simulated sequences, corroborating our model.

The files chosen for Fig 5 in the manuscript are *GSM2988386_H3K27me3_ChIPseq_Parental_RPM_rep1.bedgraph* (H3K27me3 modification data) and *GSM2988395_H3_ChIPseq_Parental_RPM_rep1.bedgraph*(control H3). Notice that we employed a naive discretization scheme, the primary purpose was to validate the regime of relevant (α, β). More sophisticated discretization schemes will not significantly change the regime. In S4 Fig, we show the population-averaged parental and the inherited and corrected daughter chromatids for the H3K4me3 data. This data from file *GSM3227882_H3K4me3_ChIPseq_Parental_RPM_rep1.bedgraph* was binarized using the same algorithm explained in S3 Fig.

Statistical Analysis of Experimental Data

To obtain the binary realizations of the H3K27me3 data from [1], we obtained the nucleosomes in the region of bp:151,495,060-165,790,665 and applied the discretization algorithm explained in S3 Fig. The data is from the HeLa cells and was available in bins of 500 bp from the database. We obtained 100 binary realizations of the mother. The average values of α and β for mother realizations were computed using Eq. (S3) and Eq. (S4) in S2 Text to yield 0.81 and 0.815 respectively. For the H3K4me3 data represented in S4 Fig the mean α and β values of the sequences binarized in the region are 0.723 and 0.979 respectively.

Each of the mother sequences were ANDed with a IID binary sequence (generated by independent flips of an unbiased coin) to produce 100 daughters. They were corrected with the threshold-k filling algorithm with different values of k_t. The results are plotted in Fig 5 of the manuscript.

Simulations in the context of other computational models

We show that if the parental population of cells have two stable states (bistable), the inherited population also will have two stable states. This can be the ensemble interpretation of bistable states similar to the work of Sneppen and coworkers [2–4]. We also show that if the parental population has stable modified state, it will be stably inherited like in the case of Crabtree and others [5]. The simulation results are shown in S5 Fig. For S5A Fig, we have simulated 50 mother sequences with large predominantly modified (1) and 50 mother sequences with predominantly unmodified regions (0). Each mother (of length 60) produced 100 daughter sequences. The daughter sequences were produced using the algorithm explained in S1 text. For each mother and daughter sequence, we computed the number of modified nucleosomes. The histogram of this measure is plotted for the mother and the corrected daughter sequences in S5A Fig. As can be seen from the figure, both the mother and corrected daughters show a bimodal distribution.

For S5B Fig, we simulated 100 mother sequences with patterns similar to the experimentally observed sequences in the Hodges and Crabtree model [5]. The daughter sequences (100 per mother) were produced by random flipping of 1s (with probability 0.5) as explained in S1 text and were corrected using our algorithm. The corrected daughter sequences had a mean modification pattern very similar to that of the mother, as can be observed in S5B Fig.

Propagation of Error Across Generations and Block Error Computation

To understand how the error propagates from the mother chromatin to the daughters over several replication cycles, we simulated subsequent cycles of replication from an original set of mother chromatids and compared the daughter chromatids with the corresponding original mother sequence. It is important to note that we have considered the threshold-k filling algorithm to be the only correcting mechanism in these simulations without any other secondary mechanisms that may be present additionally in practice. We start with 50 mothers, each generated using $\alpha = 0.9, \beta = 0.9$ and starting with a 1. 50 daughters were generated from each mother, the first generation daughters were corrected and compared with their corresponding mothers. Each of these 2500 daughters from this generation were tagged with their mother's identity for traceability of the lineage . From the next generation onwards, the mothers were chosen randomly from the previous generation daughters (50 were picked randomly from 2500). Each of these 50 mothers produced 50 daughters which retained the tag of the original mothers. These were corrected and we computed the "error" in two different ways:

(1) The bit error rate (BER) as discussed in the main manuscript where each bit (nucleosome) of the daughter is compared with the corresponding bit of the original mother and the BER is computed.

(2) Block error: To account for the possibility that biological systems may be computing the similarity in patterns with the mother chromatin by comparing blocks of histone modifications, we computed the block error rate as follows: We averaged the bits over blocks of various sizes in a sliding window in both the mother chromatin and the corrected daughter chromatin. That is, in each array $\mathbf{M}$, starting from 1, a block of size b nucleosomes were considered. The average of b bits were computed as

$$\bar{m}_i = \frac{1}{b}\sum_{j=i}^{i+b} m_j \tag{S1}$$

This process was repeated for the corrected daughter. The block averaged sequences were then used for computation of error using Eq. (3) in the manuscript. We have plotted the results having both the errors in S6A Fig. When block size is 1, we get BER. One can observe that as the block size increases, the error decreases monotonically. Both kinds of errors are also plotted for different generations. Since any non-zero error would propagate, here too, the error increases over generations. However, the error propagation is smaller as we increase the block size.

We also compute the pattern of modifications for H3K27me3 for different generations. The result is shown in S6B Fig. One can observe that the mean modification pattern is similar across generations. The data is obtained from the HeLa cells (from [1] in the region of Chr1 : bp:151,495,060-151,995,060).

[1] Reverón-Gómez N, González-Aguilera C, Stewart-Morgan KR, Petryk N, Flury V, Graziano S, Johansen JV, Jakobsen JS, Alabert C, Groth A. 2018 Accurate recycling of parental histones reproduces the histone modification landscape during DNA replication. *Molecular Cell* **72**, 239–249.

[2] Dodd IB, Micheelsen MA, Sneppen K, Thon G. 2007 Theoretical analysis of epigenetic cell memory by nucleosome modification. *Cell* **129**, 813–822.

[3] Sneppen K, Ringrose L. 2019 Theoretical analysis of Polycomb-Trithorax systems predicts that poised chromatin is bistable and not bivalent. *Nature communications* **10**, 1–18.

[4] Berry S, Dean C, Howard M. 2017 Slow chromatin dynamics allow polycomb target genes to filter fluctuations in transcription factor activity. *Cell systems* **4**, 445–457.

[5] Hodges C, Crabtree GR. 2012 Dynamics of inherently bounded histone modification domains. *Proceedings of the National Academy of Sciences* **109**, 13296–13301.
